# RUNX3 pathway signature predicts clinical benefits of immune checkpoint inhibition plus tyrosine kinase inhibition in advanced renal cell carcinoma

**DOI:** 10.1186/s12894-023-01356-w

**Published:** 2024-01-03

**Authors:** Jiajun Wang, Sihong Zhang, Ying Wang, Yanjun Zhu, Xianglai Xu, Jianming Guo

**Affiliations:** 1 Department of Urology, Zhongshan Hospital, Fudan University, No.180 Fenglin Road, Shanghai, 200032 China; 2 Department of Critical Care Medicine, Zhongshan Hospital, Fudan University, Shanghai, 200032 China

**Keywords:** Renal cell carcinoma, Runt-related transcription factor 3, Immune checkpoint inhibition plus tyrosine kinase inhibition, Immune evasion

## Abstract

**Background:**

Checkpoint inhibitor immunotherapy plus tyrosine kinase inhibitor (IO/TKI) have been recently recommended as standard first-line therapy for advanced renal cell carcinoma, while no clinical-available biomarker has been applied. This study aimed to investigate the associations between RUNX3 pathway signature and IO/TKI benefits in renal cell carcinoma (RCC).

**Methods:**

Two IO/TKI cohorts (ZS-MRCC, JAVELIN-101) and one high-risk localized RCC cohort (ZS-HRRCC) were included. All samples were evaluated by RNA-sequencing, and RUNX Family Transcription Factor 3 (RUNX3) pathway were determined by single sample gene set enrichment analysis. Flow cytometry were applied for immune cell infiltration and function.

**Results:**

RUNX3 signature was elevated in RCC samples, compared non-tumor tissues (P < 0.001). High-RUNX3 signature was associated with shorter progression-free survival (PFS) in both IO/TKI cohorts (ZS-MRCC cohort, P = 0.025; JAVELIN-101 cohort, P = 0.019). RUNX3 signature also predicted IO/TKI benefit in advanced RCC, compared with TKI monotherapy (interaction p = 0.027). RUNX3 signature was associated with decreased number of GZMB + CD8 + T cells (Spearman’s ρ=-0.42, P = 0.006), and increased number of PD1 + CD8 + T cells (Spearman’s ρ = 0.29, P = 0.072). Moreover, the integration of RUNX3 signature and GZMB expression showed predictive potential for TKI/IO (log-rank P < 0.001). In addition, the predictive value of RUNX3 signature for IO/TKI benefit was restricted in SETD2-wild type patients (log-rank P < 0.001). Finally, a risk score was established by random forest for IO/TKI benefit, showing remarkable predictive potency (Log-rank P < 0.001).

**Conclusions:**

RUNX3 pathway signature could be a potential predictive biomarker for IO/TKI treatment in advanced RCC, for both prognosis and treatment selection between IO/TKI and TKI monotherapy.

**Supplementary Information:**

The online version contains supplementary material available at 10.1186/s12894-023-01356-w.

## Introduction

Metastatic renal cell carcinoma (RCC) is still incurable [1], although its treatment strategies have evolved significantly [2]. Recently, the combination of immunotherapy (IO) and tyrosine kinase inhibitors (TKIs) have exhibited outstanding efficacies [3–5], and IO/TKI combinations have been recommended as first-line therapies for metastatic RCC by the European Association of Urology (EAU) guideline [6]. However, no available biomarker has been applied clinically. This study aimed to discover biomarkers for IO/TKI benefit in RCC.

The runt-related transcription factor-3 (RUNX3) gene belongs to the evolutionarily conserved runt domain family of transcription factors [7]. RUNX3 acts as a developmental regulator, and it has been generally described as a tumor suppressor [8–11]. The double-sided role of RUNX3 was also reported in pancreatic ductal adenocarcinoma, both as a tumor suppressor by suppressing tumor cell proliferation, and as a tumor promoter by orchestrating a protein-secreting program supporting tumor metastasis [12]. In RCC, RUNX3 was upregulated in tumor tissues, compared with normal tissues according to data from The Cancer Genome Atlas (TCGA) [13]. Interestingly, high level of RUNX3 methylation was found to be connected with poor prognosis in RCC [14]. More importantly, RUNX3 was found to suppress tumor growth, migration, angiogenesis and metastasis of RCC [15, 16]. In summary, RUNX3 could act as a major regulator of tumor progression and metastasis.

RUNX3 is linked with T-lineage lymphocyte development as a developmental regulator [17, 18]. Runx3 also drives progenitor to T-lineage transcriptome conversion in mouse T cell commitment [19]. These findings imply the potential role of RUNX3 in inflammatory regulation. Importantly, Runx3 was found to drive a CD8 + T cell tissue residency program [20]. In tumors, Runx3 was also found as a critical regulator to program CD8 + T cell residency [21]. However, since IO/TKI therapy has just recently emerged, whether RUNX3 could regulate anti-tumor immunity, and the correlation between RUNX3 and IO/TKI benefits, has not been clarified yet.

In this study, we aimed to build an integrative signature to describe the activation status of RUNX3 pathway based on RNA-sequencing data. The predictive value of the RUNX3 pathway signature was assessed for IO/TKI therapy, as well as its correlation with tumor microenvironment components, especially with CD8^+^ T cells.

## Materials and methods

### Study cohorts and data collection

Two IO/TKI cohorts (ZS-MRCC, JAVELIN-101) and one high-risk localized RCC cohort (ZS-HRRCC) were included in the study. Another cohort from public database, TCGA-KIRC cohort, was also applied in the study.

In the ZS-MRCC cohort, 51 metastatic RCC patients treated by IO/TKI therapy were enrolled, from January 2017 to December 2020. Inclusion and exclusion criteria were listed in Supplementary Table S1. Six patients were excluded due to sample unavailability or loss of follow-up. Finally, clinical information, pathologic information, treatment response, and survival information of 45 patients was retrospectively obtained from medical records and listed in Supplementary Table S2. The RECIST 1.1 criteria were utilized to define therapeutic response and disease progression [22].

In the ZS-HRRCC cohort, 43 patients with high-risk localized RCC treated by radical nephrectomy were enrolled, from Jan 2020 to Dec 2021. Inclusion and exclusion criteria were also listed in Supplementary Table S1. Three patients were excluded due to sample unavailability or not reaching sample quality control standards. Clinical and pathologic information of the rest 40 patients was retrospectively obtained from medical records.

In the JAVELIN-101 cohort, 726 patients of advanced RCC treated by either IO/TKI (avelumab + axitinib, n = 354) or TKI monotherapy (sunitinib, n = 372) were enrolled [3]. Inclusion and exclusion criteria were described in the previous study [3]. Clinical, pathologic, somatic mutation, RNA-sequencing, and follow-up information were all acquired from the previous studies by Robert J. Motzer et al. [3, 23].

In the TCGA-KIRC cohort, 530 patients with clear cell RCC were enrolled [24]. Clinical, pathologic, RNA-sequencing, somatic mutation, and follow-up data were downloaded from the UCSC xena browser (https://xena.ucsc.edu/) [24].

### RNA-sequencing and data processing

The MagBeads Total RNA Extraction Kit (MAJORIVD) was used to isolate total RNA. Total RNA was further purified via RNAClean XP Kit (Beckman Coulter) and RNase-Free DNase Set (QIAGEN), according to the manufacturer’s instructions. Library construction and sequencing were performed by Shanghai Biotechnology Corp (Shanghai, China). VAHTS Universal V6 RNA-sequencing Library Prep Kit for Illumina (Vazyme) was utilized for RNA library preparation and NovaSeq 6000 equipment (Illumina) for sequencing. Data of RNA-sequencing was further standardized to read count and FPKM.

### Flow cytometry

Peripheral blood samples were collected before surgery, and preserved in heparinized tubes at 4 °C until experimentation (within 2 h). After adding RBC Lysis Buffer.

(Thermo Fisher Scientific), white blood cells were extracted. Surgically-resected RCC samples were freshly minced and digested with collagenase IV (Sigma) and DNase I (Sigma) at 37 °C, strained through a 70-µm strainer, and then treated with RBC lysis buffer (Thermo Fisher Scientific). After Fc receptors blockade, peripheral white blood cells, or single cell suspensions, were stained at 4 °C with fluorescently labeled membrane marker antibodies for 30 min. Intracellular proteins were stained with appropriate antibodies after being dissolved in Intracellular Fixation & Permeabilization Buffer (Thermo Fisher Scientific). Peripheral white blood cells, or single cell suspensions, were stained with antibodies labeled with fluorochrome and maintained with cell staining buffer. Flowjo v10.0 was used for analyzing BD LSRFortessaTM X-20 (BD Biosciences) FACS data (Tree Star). Supplementary Table S3 provides information about antibodies in detail.

### In silico approaches

All analysis approaches were performed on the platform of R software (https://www.r-project.org/). Single sample gene set enrichment analysis (ssGSEA) was performed via “GSVA” package to calculate RUNX3 pathway signature for each sample [25]. The genes for RUNX3 pathway signature, listed in Supplementary Table S4, were obtained from the gene set “regulation of RUNX3 expression and activity pathway” in the REACTOME dataset, as specified in MSigDB [26].

Cox regression and Kaplan-Meier analyses were performed by “survival” and “survminer” packages of R software. The Forest plots were realized by “forestplot” package of R software. The waterfall plot was plotted by “ComplexHeatmap” and “ggplot2” packages of R software. The random forest model construction was performed by “randomForestSRC” and “ggRandomForests” packages of R software.

### Statistical analyses

Wilcoxon signed ranks test, or Kruskal-Wallis H test, were used to compare continuous variables between groups. Chi-square test, Fisher’s exact analysis or Cochran-Mantel-Haenszel test were applied for categorical variables, inf appropriate. Correlational analysis was performed by Spearman’s analysis. High- and low-expression groups were generally divided by median for continuous variables. Kaplan-Meier analysis with log-rank regression, and Cox proportional hazard models, were used for survival analysis. All data procession was performed by R software (version 4.0.2; https://www.r-project.org/). Two-tailed P value < 0.05 was set as statistically significant. The level of precision was according to the guidelines by Assel et al. [27].

## Results

### Elevated expression of RUNX3 pathway signature in advanced, high-grade RCC

**Fig. 1:**
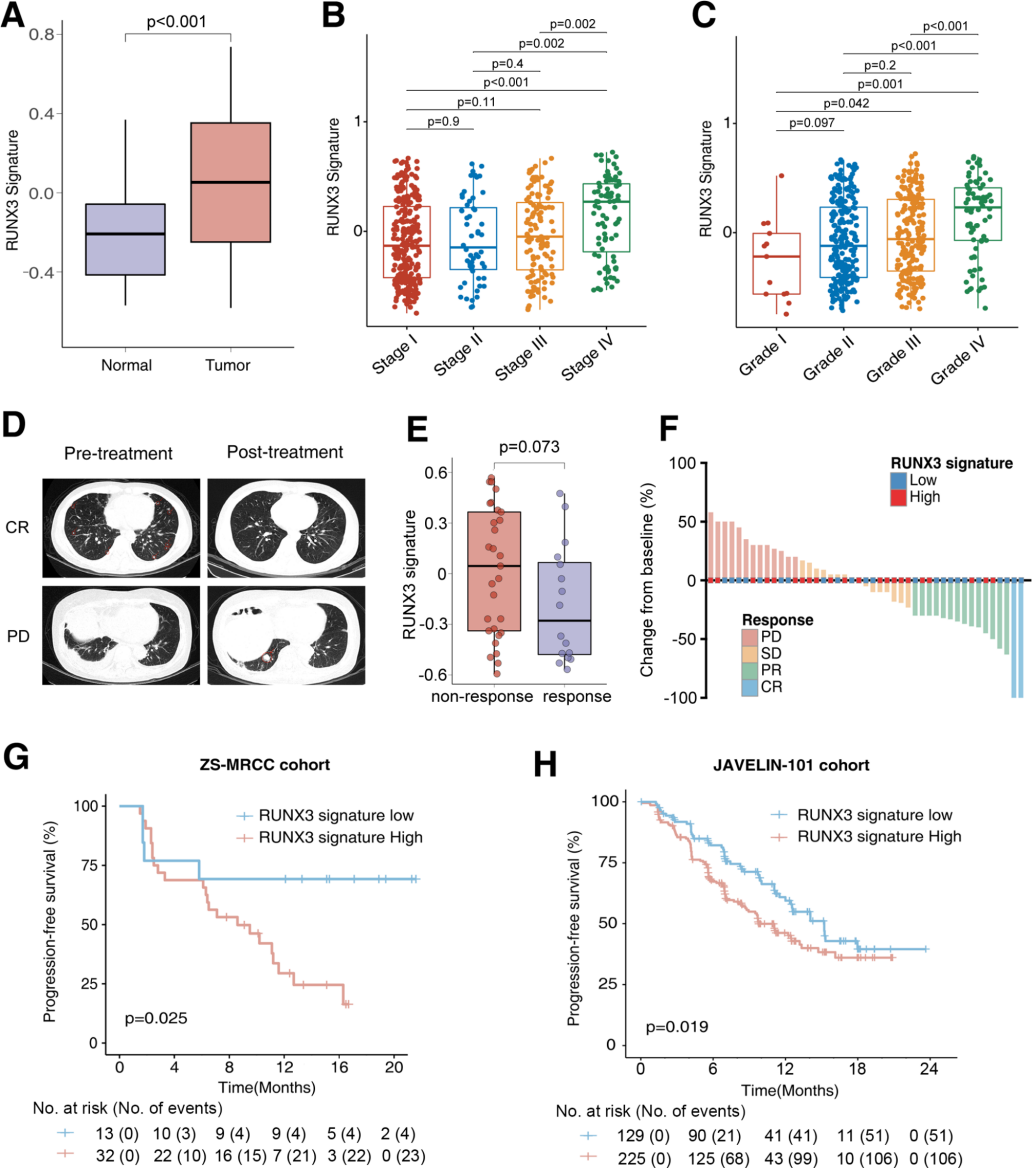
RUNX3 pathway signature associated with IO/TKI therapy response and prognosis in RCC. (**A**) Expression of RUNX3 pathway signature in tumor tissues and peritumoral non-tumor tissues. P value, Wilcoxon rank-sum test. (**B-C**) Expression of RUNX3 pathway signature in RCC of different (**B**) TNM stages and (**C**) ISUP grades. P values, Kruskal-Wallis H test. (**D**) Pre- and post-treatment computed tomography images of patients with different response after IO/TKI therapy. (**E**) RUNX3 pathway signature in responders and non-responders of IO/TKI therapy. (**F**) Best tumor shrinkage rate after IO/TKI therapy in our ZS-MRCC cohort. (**G-H**) Progression-free survival according to RUNX3 pathway signature in the (**G**) ZS-MRCC cohort and the (**H**) JAVELIN-101 cohort. P values, Kaplan-Meier analysis, and log-rank test

RUNX3 pathway signature was built by ssGSEA according to the RNA-sequencing data of each sample. RUNX3 pathway signature was elevated in tumor tissues compared with non-tumor tissues, in the TCGA-KIRC cohort (P < 0.001, Fig. 1A). RUNX3 pathway signature was elevated in advanced RCC (TNM stage IV), compared with stage I (P < 0.001), stage II (P = 0.002) and stage III (P = 0.002) samples (Fig. 1B). RUNX3 pathway signature was also associated with grade IV disease, compared with grade I (P = 0.001), grade II (P < 0.001) and grade III (P < 0.001) RCC (Fig. 1C).

### RUNX3 pathway signature associated with IO/TKI therapy response and prognosis

As shown in Fig. 1D, patients in the ZS-MRCC showed diverse therapeutic response to IO/TKI treatment. Interestingly, the RNUX3 pathway signature was elevated in non-responders of IO/TKI therapy, compared with responders, although not statistically significant (P = 0.073, Fig. 1E). After classified into high- and low-RUNX3 pathway signature groups, high-RUNX3 pathway signature was more commonly found in PD (46.2%, 6/13) or SD (68.8%, 11/16) patients, rather than PR (35.7%, 5/14) or CR (0%, 0/2) ones (Fig. 1F). Moreover, patients with a low-RUNX3 signature showed longer PFS, in both the ZS-MRCC cohort (Fig. 1G, P = 0.025) and the JAVELIN-101 cohort (Fig. 1H, P = 0.019). For multivariate Cox regression analysis, RUNX3 pathway signature was also identified as an independent factor for PFS in the ZS-MRCC cohort (HR, 3.3; 95%CI, 1.1–10; P = 0.04; Supplementary Table S5).

### RUNX3 pathway signature predicted IO/TKI benefit

**Fig. 2:**
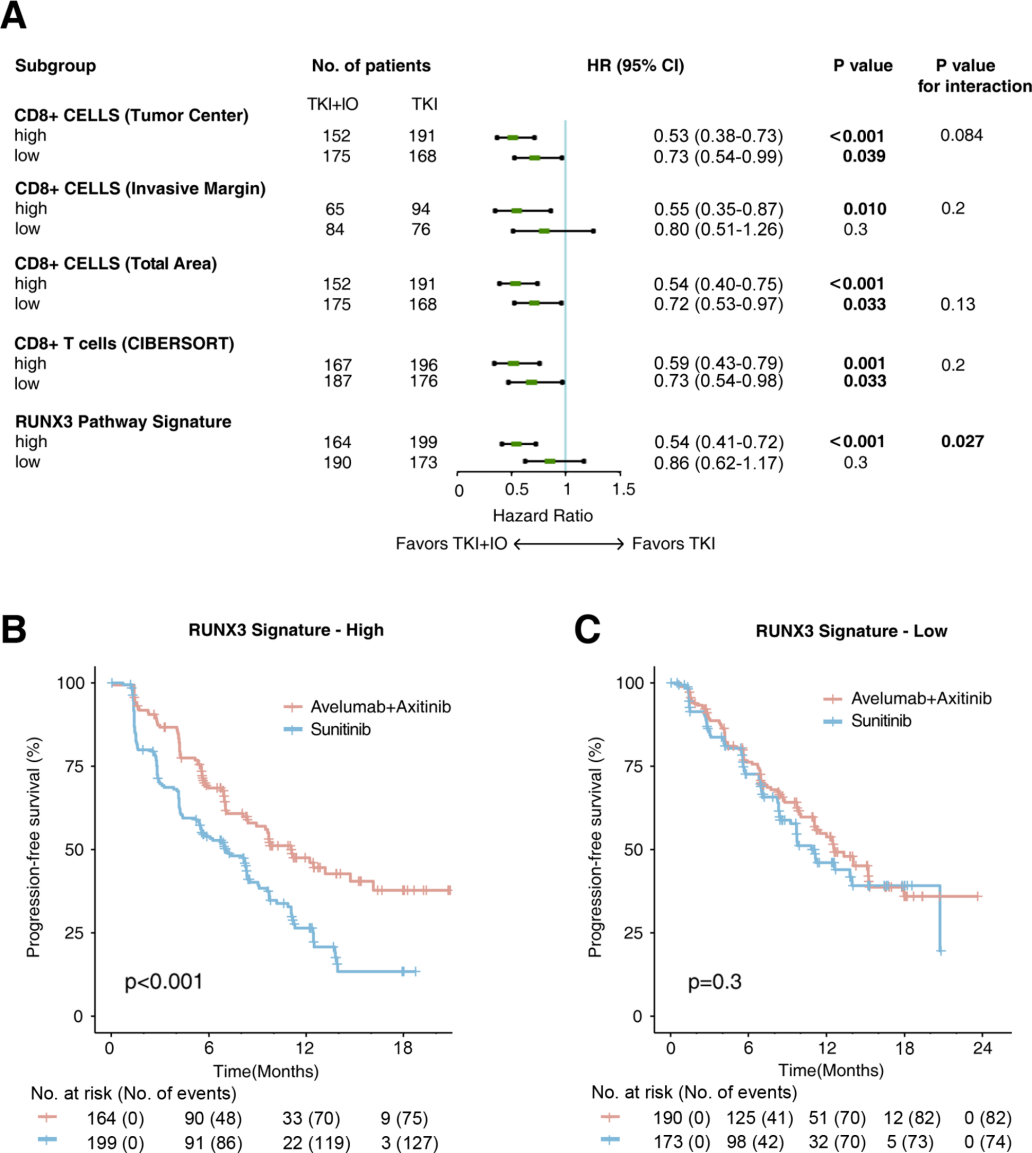
RUNX3 pathway signature predicted IO/TKI therapeutic benefit in RCC. (**A**) Survival benefit of IO/TKI versus TKI monotherapy for PFS in subgroups defined by CD8 + T cell infiltration and RUNX3 pathway signature. HR and P values, Cox regression model. (**B-C**) PFS of IO/TKI or TKI monotherapy in subgroups of (**B**) high-RUNX3 pathway signature and (**C**) low-RUNX3 pathway signature. P values, Kaplan-Meier analysis, and log-rank test

Not all patients can respond to IO/TKI therapy. As the key cytotoxic cells of TME, high infiltration groups of CD8 + T cells estimated by immunohistochemistry in different regions, or by CIBERSORT deconvolution according to RNA-sequencing data, were related with better survival under IO/TKI, (high CD8 + T cells in tumor center, HR = 0.53, P < 0.001; high CD8 + T cells in invasive margin, HR = 0.55, P = 0.010; high CD8 + T cells in total area, HR = 0.54, P < 0.001; high CD8 + T cells by CIBERSORT, HR = 0.59, P = 0.001; Fig. 2A). However, patients in the low-infiltration groups of CD8 + T cells could also show benefit under IO/TKI treatment, compared with TKI monotherapy (low CD8 + T cells in tumor center, HR = 0.73, P = 0.039; low CD8 + T cells in total area, HR = 0.72, P = 0.033; low CD8 + T cells by CIBERSORT, HR = 0.73, P = 0.033; Fig. 2A). Actually, the interaction P values between high and low CD8 + T cell infiltration groups were all statistically insignificant (Fig. 2A). These results may indicate that CD8 + T cell infiltration may be an imperfect biomarker for predicting IO/TKI therapeutic benefit versus TKI monotherapy.

Since RUNX3 pathway signature was related with IO/TKI therapy response and prognosis (Fig. 1), we further analyzed its predictive role for IO/TKI therapeutic benefit. In the high-RUNX3 pathway signature group, IO/TKI therapy showed prior survival, compared with TKI monotherapy (HR = 0.54, 95% CI 0.41–0.72, Log-rank P < 0.001, Fig. 2A and B). Meanwhile, In the low-RUNX3 pathway signature group, IO/TKI therapy showed no significant benefit (Log-rank P = 0.3, Fig. 2A C). These results indicated RUNX3 pathway signature as a potential biomarker for predicting IO/TKI therapeutic benefit versus TKI monotherapy (P for interaction = 0.027, Fig. 2A).

### CD8 + T cell dysfunction in RCC with high-RUNX3 pathway signature

**Fig. 3:**
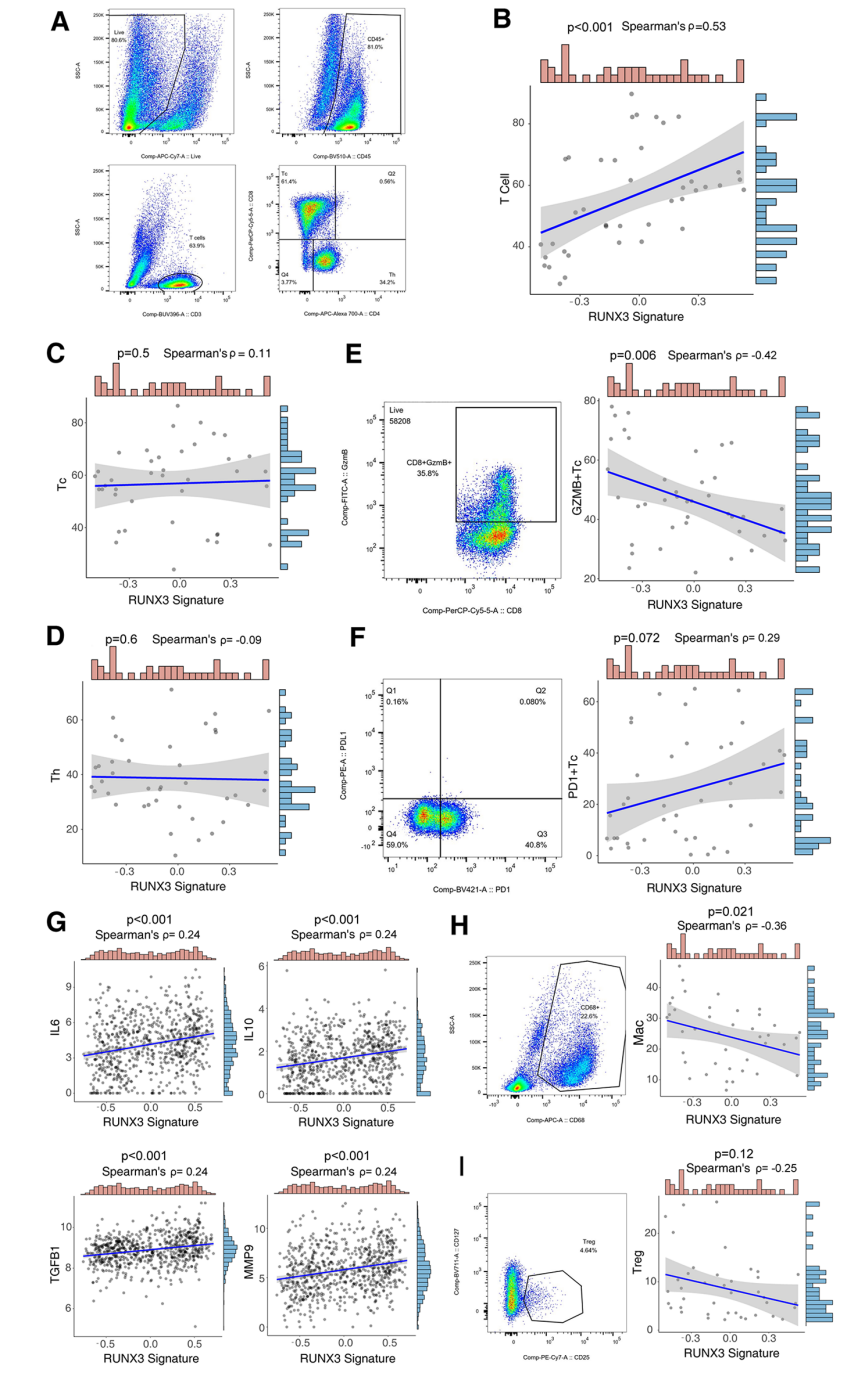
CD8 + T cell dysfunction in RCC with high-RUNX3 pathway signature (**A**) Gating strategies of T cells, CD8 + T cells and CD4 + T cells by flow cytometry in ZS-HRRCC cohort. (**B-D**) Correlation between RUNX3 pathway signature and (**B**) T cells, (**C**) CD8 + T cells and (**D**) CD4 + T cells. P values and ρ, Spearman’s correlation test. (**E-F**) Gating strategies of (**E**) GZMB + CD8 + T cells and (**F**) PD1 + CD8 + T cells by flow cytometry in ZS-HRRCC cohort, and their association with RUNX3 pathway signature. P values and ρ, Spearman’s correlation test. (**G**) Correlation between RUNX3 pathway signature and IL6, IL10, TGFB1 and MMP9 expression by RNA-sequencing. P values and ρ, Spearman’s correlation test. (**H-I**) Gating strategies of (**H**) macrophages and (**I**) regulatory T cells by flow cytometry in ZS-HRRCC cohort, and their association with RUNX3 pathway signature. P values and ρ, Spearman’s correlation test

We further performed flow cytometry on freshly-resected RCC samples from the ZS-HRRCC cohort to discover the immunologic relevance of RUNX3 pathway signature. Figure 3 A showed the gating strategy of flow cytometry for T cells, CD8 + T cells and CD4 + T cells in the ZS-HRRCC cohort. Firstly, RUNX3 pathway signature was positively associated with tumor-infiltrating lymphocytes (TILs) (Spearman’s ρ = 0.53, P < 0.001, Fig. 3B). However, neither CD8^+^ T cells (Spearman’s ρ = 0.11, P = 0.5, Fig. 3C) nor CD4^+^ T cells (Spearman’s ρ=-0.09, P = 0.6, Fig. 3D) showed association with RUNX3 pathway signature.

T cell function was regulated by regulatory components of TME, resulting in dysfunction and exhaustion, which is for immune evasion. RUNX3 pathway signature showed negative association with GZMB + CD8 + T cells (Spearman’s ρ=-0.4, P = 0.006, Fig. 3E), but positive association with PD1 + CD8 + T cells, although not statistically significant (Spearman’s ρ = 0.3, P = 0.072, Fig. 3F). However, the number of GZMB + CD4 + T cells or PD1 + CD4 + T cells showed no statistically significant association with RUNX3 pathway signature (data not shown). The results indicated CD8 + T cells dysfunction in RCC with high-RUNX3 pathway signature.

### Regulatory cytokines and cells in tumors with high-RUNX3 pathway signature

Regulatory components in TME contribute to CD8 + T cell dysfunction. In the study, high-RUNX3 pathway signature was related with gene expression of suppressive cytokines, including IL6, IL10, TGFB1 and MMP9 (Spearman’s P values < 0.001, Fig. 3G). However, high-RUNX3 pathway signature was related with sparse infiltration of macrophages (Spearman’s ρ=-0.36, P = 0.021, Fig. 3H) and regulatory T cells (Spearman’s ρ=-0.25, Fig. 3I), although not statistically significant. The results indicated the elevated content of immunosuppressive cytokines in high-RUNX3 signature RCC, which may not be reliant on infiltration of macrophages or regulatory T cells.

### Functional molecules of CD8 + T cells together with RUNX3 pathway signature to predict IO/TKI benefit

**Fig. 4:**
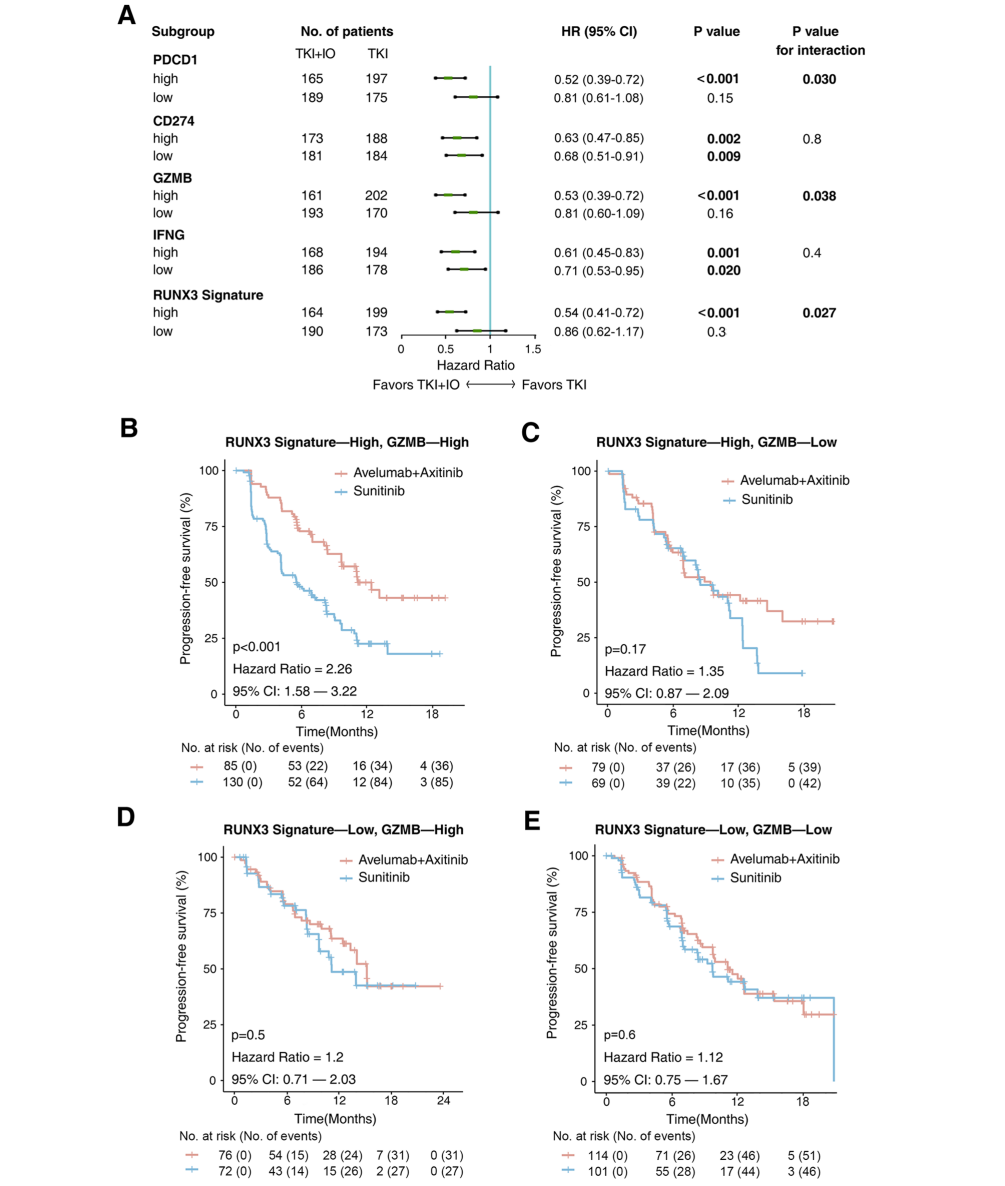
RUNX3 pathway signature together with GZMB to predict IO/TKI benefit. (**A**) Survival benefit of IO/TKI versus TKI monotherapy for PFS in subgroups defined by functional molecules of CD8 + T cells. HR and P values, Cox regression model. (**B-E**) PFS of IO/TKI or TKI monotherapy in subgroups of (**B**) high-RUNX3 signature/high-GZMB, (**C**) high-RUNX3 signature/low-GZMB, (**B**) low-RUNX3 signature/high-GZMB, (**B**) low-RUNX3 signature/low-GZMB. P values, Kaplan-Meier analysis, and log-rank test

Given the relationship between CD8 + T cell dysfunction and RUNX3 pathway signature, we further evaluated their crosstalk for predicting IO/TKI benefit. GZMB, as one of the central cytolytic molecules of CD8 + T cells, showed predictive potential (P for interaction = 0.038, Fig. 4A). In the high-GZMB subgroup, IO/TKI showed better survival, compared with TKI monotherapy (HR = 0.53, 95% CI 0.39–0.72, P < 0.001, Fig. 4A), while in the low-GZMB subgroup the IO/TKI showed no significant survival benefit (HR = 0.81, 95% CI 0.60–1.1, P = 0.4, Fig. 4A). Besides, PDCD1, as one of the exhaustion molecules of CD8 + T cells, also showed predictive potential (P for interaction = 0.030, Fig. 4A).

For more precise stratification of IO/TKI benefit, we further integrated functional molecules of CD8 + T cells and RUNX3 pathway signature. IO/TKI, versus TKI monotherapy, showed benefit only in patients with high-RUNX3 signature and high-GZMB expression (log-rank P < 0.001, Fig. 4B), rather than other subgroups (Fig. 4C and E). we also integrated other functional molecule expression, including PDCD1, CD274 and IFNG, but none of them showed better predictive value (data not shown).

### Mutations together with RUNX3 pathway signature to predict IO/TKI benefit

**Fig. 5:**
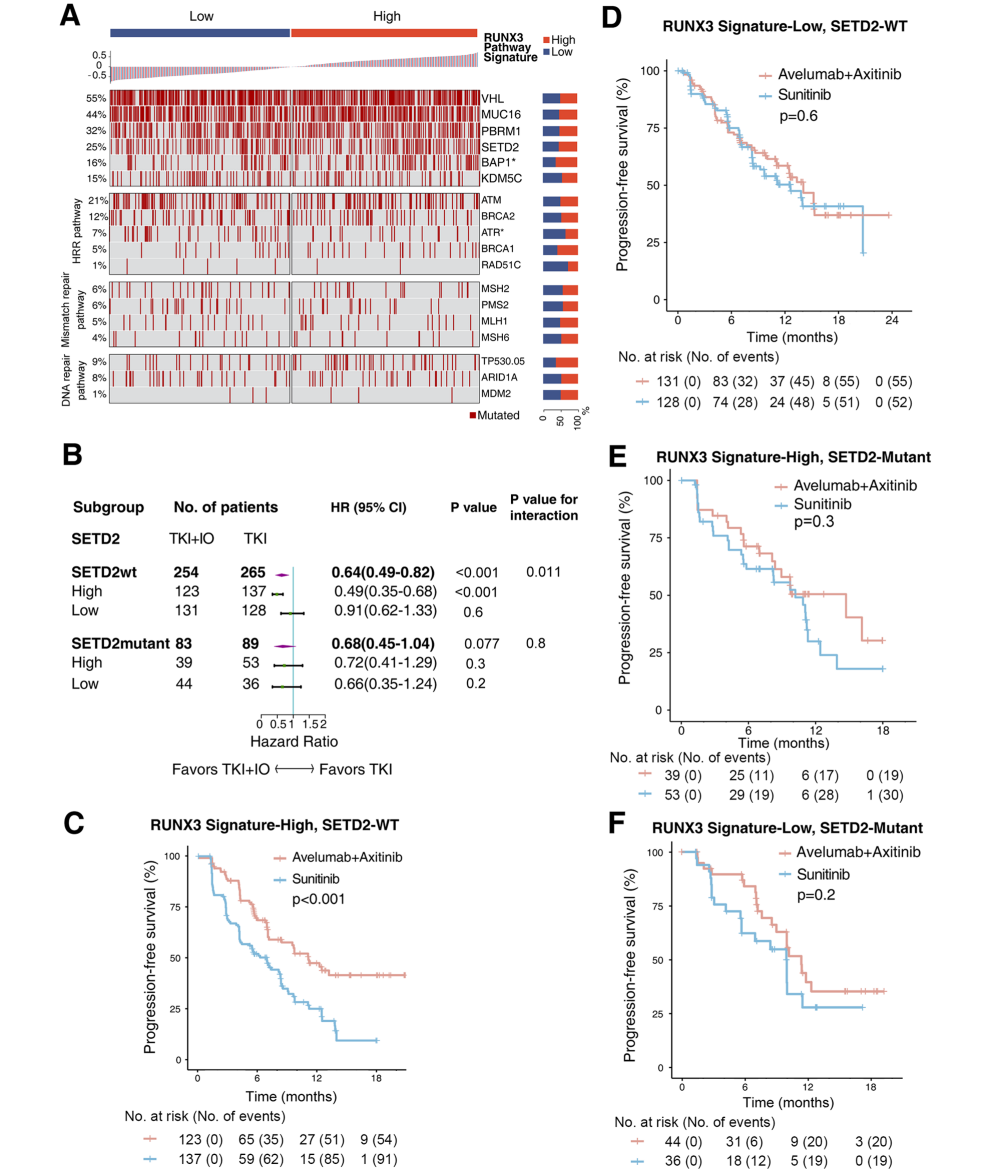
Genomic mutations together with RUNX3 pathway signature to predict IO/TKI benefit. (**A**) Waterfall plot showing genomic mutations ranked by RUNX3 signature expression. P values, Chi-square test. *, P < 0.05. (**B**) PFS benefit of IO/TKI versus TKI monotherapy defined by SETD2 mutational status and RUNX3 signature. HR and P values, Cox regression model; wt, wild type. (**C-F**) PFS of IO/TKI or TKI monotherapy in subgroups of (**C**) high-RUNX3 signature/SETD2-wt, (**D**) low-RUNX3 signature/SETD2-wild type, (**E**) high-RUNX3 signature/SETD2-mutant, (**F**) low-RUNX3 signature/SETD2-mutant. P values, Kaplan-Meier analysis, and log-rank test

Somatic mutations could impact immunotherapy response in RCC [28]. Correlation between RUNX3 pathway signature and somatic mutations in RCC was shown in Fig. 5A. In the JAVELIN-101 cohort of advanced RCC, frequent mutations included VHL (55%), MUC16(44%), PBRM1 (32%), SETD2 (25%) and BAP1 (16%). High-RUNX3 signature group showed higher frequency of BAP1 mutation, but lower rate of ATR mutation (Fig. 5A).

We also integrated somatic mutations and RUNX3 pathway signature, for more precise stratification of IO/TKI benefit. Interestingly, IO/TKI benefit was restricted in patients with wild type SETD2 and high-RUNX3 pathway signature (P < 0.001, Fig. 5B C), rather than other groups (Fig. 5D F).

### A combined prognostic risk score for IO/TKI therapy

**Fig. 6:**
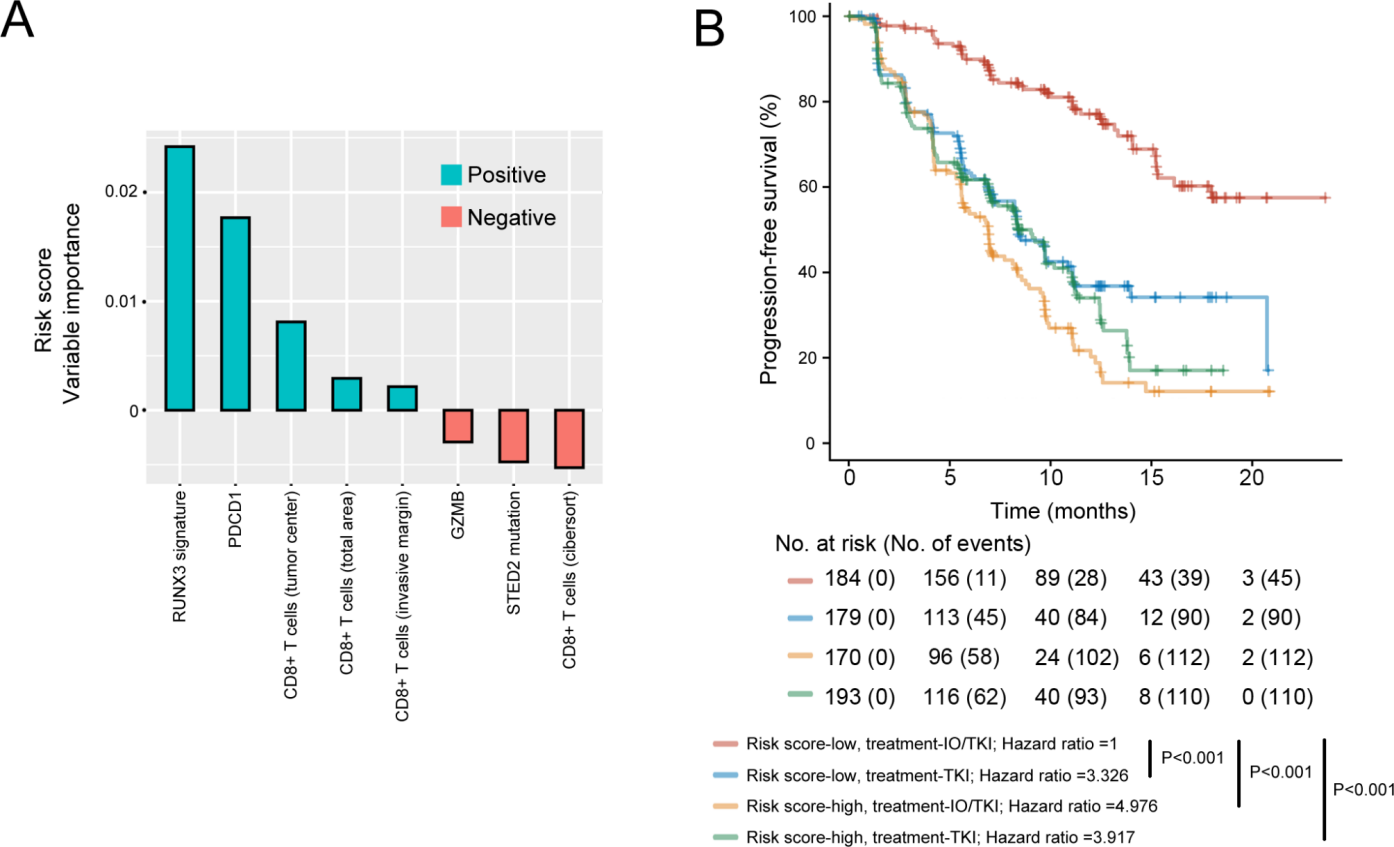
A combined risk score for IO/TKI therapy prognosis and benefit. (**A**) Variable importance of predictive parameters enrolled in the random forest risk model. (B) PFS of subgroups defined by risk score and therapeutic regimens. P value, Kaplan-Meier analysis and log-rank test

Patient selection based on tumour molecular phenotype could choose the most efficacious treatment between nivolumab, nivolumab/ipilimumab and TKI in metastatic RCC [29]. Finally, we intended to build a risk model for treatment selection between IO/TKI and TKI monotherapy in RCC. Predictive parameters previously described in the study, including RUNX3 signature, CD8 + T cell infiltration, GZMB, PDCD1 and SETD2 mutation were enrolled in the risk model. After model construction by random forest algorism for machine learning, RUNX3 pathway signature showed the most outstanding positive contribution for the model (Fig. 6A). In patients with low-risk score, IO/TKI therapy led to better survival, compared with TKI monotherapy (P < 0.001, Fig. 6B). However, in patients with high-risk score IO/TKI therapy did not lead to significant survival benefit (Fig. 6B).

## Discussion

The RUNX3 pathway act as a major regulator of tumor progression and metastasis, hence influencing patients’ survival [12–16]. In this study, a RUNX3 pathway signature was calculated by ssGSEA to define the regulation and activation of RUNX3 pathway. RUNX3 pathway signature was found associated with response under IO/TKI therapy and could predict IO/TKI benefit, compared with TKI monotherapy. RUNX3 pathway signature was also found associated with CD8 + T cell dysfunction and immunosuppressive cytokines in TME. Lastly, a risk score combining RUNX3 pathway signature, somatic mutations, CD8 + T cell infiltration and immune checkpoints showed excellent prognostic and predictive potential for IO/TKI efficacy.

RUNX3 gene belongs to the runt domain family, which is highly conserved during evolution [7]. Usually, RUNX3 acts as a tumor suppressor gene in various human neoplasms [8–11]. In RCC, RUNX3 was found to suppress tumor growth, migration, angiogenesis and metastasis [15, 16]. However, in the current study, elevated expression of RUNX3 pathway signature was found in RCC tissues, compared with normal tissues (Fig. 1A). Additionally, RUNX3 pathway signature was also elevated in high-stage, high-grade RCC (Fig. 1B C). Recently, double-sided role of RUNX3 as both a tumor suppressor and as a tumor promoter has just been discovered in pancreatic cancer [12]. Accordingly, we suppose RUNX3 might be more complicated than just tumor-suppressor gene. The hypothesis still needs further mechanistic explorations.

RUNX3 is a regulator for T cell development [17, 18], and was found to drive a CD8 + T cell tissue residency program [20]. In tumor microenvironment, Runx3 was also found as a critical regulator of CD8 + T cell residency [21]. However, whether RUNX3 could regulate anti-tumor immunity, and the correlation between RUNX3 and IO/TKI benefits, has not been clarified yet. In the current study, RUNX3 pathway signature was found associated with IO/TKI response and survival (Fig. 1E H). More importantly, RUNX3 pathway signature predicted IO/TKI benefit, versus TKI monotherapy (Fig. 2). Additionally, RUNX3 pathway signature correlated with CD8 + T cell dysfunction, characterized by decreased GZMB expression measured by flow cytometry (Fig. 3E). The results led to a point that RUNX3 pathway could regulate CD8 + T cell function in TME, thus impact tumor microenvironment and IO/TKI benefit. RUNX3 is required for the synthesis of effector molecules in cytotoxic CD8 + T cells, and cultured mouse Runx3-/- Cd8 + T cells produced considerably less GZMB than wild-type cells [30]. However, the molecular mechanism behind the dysfunction of CD8^+^ T cells is poorly understood at present.

Both IO/TKI and TKI monotherapy are first-line recommendations by recently updated guidelines of RCC [31]. Several clinical trials revealed the superior role of IO/TKI versus TKI monotherapy in the general population [3, 32, 33]. Nevertheless, response and survival under IO/TKI could vary in each patient (Fig. 1D). Recently, a biomarker-driven phase 2 trial, BIONIKK study, showed patient selection based on tumour molecular phenotype could choose the most efficacious treatment between IO and TKI in metastatic RCC [29]. However, no similar study has been performed for IO/TKI versus TKI monotherapy so far in RCC. In our research, we identified RUNX3 pathway signature as a novel predictor of IO/TKI response and PFS (Fig. 1F H). Moreover, a novel risk score combining RUNX3 pathway signature, somatic mutations, CD8 + T cell infiltration and immune checkpoints showed excellent predictive potential for IO/TKI efficacy (Fig. 6). Considering the retrospective design of the study, the results still need to be validated in prospective, larger studies. However, the study still revealed that patient selection based on RNA-sequencing could choose the better therapy, between IO/TKI and TKI, in metastatic RCC.

As a chromatin remodeling gene, SETD2 mutation was found to be associated with IO therapeutic response in a pan-cancer study [34]. However, although SETD2 mutation is frequently occurred in RCC, no significant correlation was found between SETD2 mutation and IO therapeutic response in RCC [28]. In the current study, IO/TKI showed significant benefit, compared with TKI, only in SETD2-wild type patients (HR 0.64, 95%CI 0.49–0.82, P < 0.001, Fig. 5B). Besides, even in SETD2-wild type patients, only those with high RUNX3 pathway signature could benefit from adding IO to TKI therapy (HR 0.49, 95%CI 0.35–0.68, P value for interaction = 0.011). According to the results, SETD2 mutational status and RUNX3 pathway might work together for predicting TKI + IO benefit. However, no current study has reported the regulation of RUNX3 by SETD2, which need to be identified by further studies.

The study still has several limitations. The retrospective methodology and relatively small sample size may lead to bias. This is due to IO/TKI has just been recommended as first-line therapy recently. However, further prospective validation study in larger cohorts is also ongoing. Additionally, the mechanism of the relationship between RUNX3 pathway, T cell function, and immunotherapy remains obscure, which should be discovered in the future Moreover, whether tumor biopsies could also be applied for assessing RUNX3 pathway for predicting IO/TKI benefit also needs future research.

## Conclusions

RUNX3 pathway signature could be a potential predictive biomarker for IO/TKI treatment in advanced RCC, for both prognosis and treatment selection between IO/TKI and TKI monotherapy. RUNX3 pathway was related with CD8 + T cell dysfunction in tumor microenvironment.

### Electronic supplementary material

Below is the link to the electronic supplementary material.

**Table S1**. Inclusion and exclusion criteria for ZS-MRCC and ZS-HRRCC cohorts.

**Table S2**. Baseline demographic and clinical characteristics of the ZS-MRCC cohort.

**Table S3**. Information of antibodies for flow cytometry.

**Table S4**. Genes in the REACTOME_REGULATION_OF_RUNX3_EXPRESSION_AND_ACTIVITY gene set.

**Table S5**. Multivariate Cox regression analysis for progression-free survival in the ZS-MRCC cohort.

## Data Availability

The datasets used and/or analyzed during the current study available from the corresponding author on reasonable request.
