# Supplementary material for: RUNX3 pathway signature predicts clinical benefits of immune checkpoint inhibition plus tyrosine kinase inhibition in advanced renal cell carcinoma
Source: BMC Urol. 2024 Jan 3;24:8. doi: 10.1186/s12894-023-01356-w (PMC10765845; doi:10.1186/s12894-023-01356-w)
Supplement: Supplementary file 2 — Table S2. Baseline demographic and clinical characteristics of the ZS-MRCC cohort. [file 12894_2023_1356_MOESM2_ESM.doc]

| Table S2. Baseline demographic and clinical characteristics of the ZS-MRCC cohort. | |
| --- | --- |
|  | ZS-MRCC cohort, n=45 |
| Age, median (range) | 62 (18-79) |
| Gender |  |
| Male | 25 (55.6%) |
| Female | 20 (44.4%) |
| Nephrectomy | 45 (100.0%) |
| Histology |  |
| Clear cell | 31 (68.9%) |
| Papillary | 5 (11.1%) |
| Chromophobe | 1 (2.2%) |
| Xp11.2 Translocation | 3 (6.7%) |
| Sarcomatoid variants | 3 (6.7%) |
| Unclassified | 2 (4.4%) |
| ISUP grade |  |
| II | 22 (48.9%) |
| III | 15 (33.3%) |
| IV | 6 (13.3%) |
| Regimens |  |
| Axitinib/Tislelizumab | 22 (48.9%) |
| Axitinib/Sintilimab | 8 (17.8%) |
| Lenvatinib/Pembrolizumab | 15 (33.3) |
| Line of therapy* |  |
| First-line | 20 (44.4%) |
| Second-line | 13 (28.9%) |
| Third-line | 12 (26.7%) |
| IMDC risk group |  |
| Favorable | 8 (17.8%) |
| Intermediate | 31 (68.9%) |
| Poor | 6 (13.3%) |
| Best response |  |
| Complete response | 2 (4.4%) |
| Partial response | 14 (31.1%) |
| Stable disease | 16 (35.6%) |
| Progressive disease | 13 (28.9%) |
| RUNX3 pathway signature |  |
| Low | 23 (51.1%) |
| High | 22 (48.9%) |
| * Two patients were not classified for ISUP grade because of unclassified histology. | |
