# Supplementary material for: RUNX3 pathway signature predicts clinical benefits of immune checkpoint inhibition plus tyrosine kinase inhibition in advanced renal cell carcinoma
Source: BMC Urol. 2024 Jan 3;24:8. doi: 10.1186/s12894-023-01356-w (PMC10765845; doi:10.1186/s12894-023-01356-w)
Supplement: Supplementary file 3 — Table S3. Information of antibodies for flow cytometry. [file 12894_2023_1356_MOESM3_ESM.doc]

| Table S3. Information of antibodies for flow cytometry | | | | | |
| --- | --- | --- | --- | --- | --- |
| Name | Company | Catalogue No. | Application | Diluted |  |
| Fixable Viability Dye, eFluor780 | ThermoFisher Scientific | 65-0865-18 | Flow cytometry | 1:200 |  |
| Mouse anti-human CD45, BV510 | BioLegend | 368526 | Flow cytometry | 1:100 |  |
| Mouse anti-human CD68, AF647 | BioLegend | 333820 | Flow cytometry | 1:100 |  |
| Mouse anti-human CD3, BUV395 | BD Biosciences | 564001 | Flow cytometry | 1:100 |  |
| Mouse anti-human CD4, AF700 | ThermoFisher Scientific | 56-0049-42 | Flow cytometry | 1:100 |  |
| Mouse anti-human CD8, PerCP-Cy5.5 | ThermoFisher Scientific | 45-0088-42 | Flow cytometry | 1:100 |  |
| Mouse anti-human CD279, BV421 | BD Biosciences | 564323 | Flow cytometry | 1:100 |  |
| Mouse anti-human CD274, PE | ThermoFisher Scientific | 12-5983-42 | Flow cytometry | 1:100 |  |
| Mouse anti-human CD25, PE-Cy7 | ThermoFisher Scientific | 25-0259-42 | Flow cytometry | 1:100 |  |
| Mouse anti-human CD127, BV711 | BD Biosciences | 563165 | Flow cytometry | 1:100 |  |
| Mouse anti-human/mouse Granzyme B, FITC | BioLegend | 515403 | Flow cytometry | 1:200 |  |
