# Supplementary material for: RUNX3 pathway signature predicts clinical benefits of immune checkpoint inhibition plus tyrosine kinase inhibition in advanced renal cell carcinoma
Source: BMC Urol. 2024 Jan 3;24:8. doi: 10.1186/s12894-023-01356-w (PMC10765845; doi:10.1186/s12894-023-01356-w)
Supplement: Supplementary file 4 — Table S4. Genes in the REACTOME_REGULATION_OF_RUNX3_EXPRESSION_AND_ACTIVITY gene set. [file 12894_2023_1356_MOESM4_ESM.doc]

| Table S4. Genes in the REACTOME_REGULATION_OF_RUNX3_EXPRESSION_AND_ACTIVITY gene set. | | | | |
| --- | --- | --- | --- | --- |
| CBFB | PSMB1 | PSMC2 | PSMD2 | PSMF1 |
| CDKN2A | PSMB10 | PSMC3 | PSMD3 | RPS27A |
| EP300 | PSMB2 | PSMC4 | PSMD4 | RUNX3 |
| MDM2 | PSMB3 | PSMC5 | PSMD5 | SEM1 |
| PSMA1 | PSMB4 | PSMC6 | PSMD6 | SMURF1 |
| PSMA2 | PSMB5 | PSMD1 | PSMD7 | SMURF2 |
| PSMA3 | PSMB6 | PSMD10 | PSMD8 | SRC |
| PSMA4 | PSMB7 | PSMD11 | PSMD9 | TGFB1 |
| PSMA5 | PSMB8 | PSMD12 | PSME1 | UBA52 |
| PSMA6 | PSMB9 | PSMD13 | PSME2 | UBB |
| PSMA7 | PSMC1 | PSMD14 | PSME3 | UBC |
| CBFB | PSMB1 | PSMC2 | PSMD2 | PSMF1 |
| CDKN2A | PSMB10 | PSMC3 | PSMD3 | RPS27A |
| EP300 | PSMB2 | PSMC4 | PSMD4 | RUNX3 |
| MDM2 | PSMB3 | PSMC5 | PSMD5 | SEM1 |
