# Supplementary material for: RUNX3 pathway signature predicts clinical benefits of immune checkpoint inhibition plus tyrosine kinase inhibition in advanced renal cell carcinoma
Source: BMC Urol. 2024 Jan 3;24:8. doi: 10.1186/s12894-023-01356-w (PMC10765845; doi:10.1186/s12894-023-01356-w)
Supplement: Supplementary file 5 — Table S5. Multivariate Cox regression analysis for progression-free survival in the ZS-MRCC cohort. [file 12894_2023_1356_MOESM5_ESM.doc]

| Table S5. Multivariate Cox regression analysis for progression-free survival in the ZS-MRCC cohort. | | |
| --- | --- | --- |
| Variables | ZS-MRCC cohort (n= 45) | |
|  | HR (95%CI) † | *p* value † |
| Histology |  | 0.5 |
| Non-clear cell *vs* clear cell type | 0.71 (0.28-1.8) |  |
| IMDC risk group |  | 0.5 |
| Intermediate/poor *vs* favorable | 1.5 (0.48-4.5) |  |
| ISUP grade |  | 0.15 |
| II | 1 [Reference] |  |
| III | 0.54 (0.22-1.4) | 0.19 |
| IV | 1.9 (0.60-5.8) | 0.3 |
| RUNX3 pathway signature |  | **0.04** |
| High *vs* low | 3.3 (1.1-10) |  |
| Abbreviations: HR, Hazard ratio; CI, confidence interval; IMDC, International mRCC Database Consortium.  †Data obtained from multivariate Cox regression model. *p* value<0.05 were marked in bold font. | | |
